# Supplementary material for: The impact of castration on physiological responses to exertional heat stroke in mice
Source: PLoS One. 2022 Oct 13;17(10):e0275715. doi: 10.1371/journal.pone.0275715 (PMC9560521; doi:10.1371/journal.pone.0275715)
Supplement: Attachment — Submitted filename: Review Summary r1.pdf [file pone.0275715.s001.pdf]

Review Summary

In this manuscript, the authors detail an experiment intended to test whether castration, and therefore the removal of testosterone, from male mice affects the physiological response to EHS. Contrary to previous findings, no substantial differences were observed in the castrated mice compared to the control (EHS) mice. While the sample size is small, I believe the experimental design is adequate, and the conclusions/discussion are in concordance with the reported results. However, I have detailed a few comments that need to be addressed below.

Major Comments

1. The results section currently lacks any display of the p-values that the hypothesis tests are based upon or effect sizes. I would strongly recommend the inclusion of both in the results. In particular, exact/precise p-values (e.g., $p = 0.048$ not $p < 0.05$) would be preferrable (even on the figures) since this is considered standard in physiology research https://journals.physiology.org/doi/full/10.1152/advan.00022.2007
    a. As for effect sizes, I would recommend eta-squared for the Kruskal-Wallis and the rank-biserial correlation coefficient for the Wilcoxon sum-rank tests
    b. For the Kruskal-Wallis effect size; $\eta^2 = (H - k + 1)/(n - k)$, wherein H refers to the test statistic from the Kruska-Wallis tests, k is the number of groups, and n is the total sample size.
    c. For the Wilcoxon sum rank effect size see this article by Kirby for a variety of formulaic approaches. https://journals.sagepub.com/doi/full/10.2466/11.IT.3.1
2. In the results, it is unclear which tests are based on an ANOVA/t-test or the non-parametric tests. Statistically speaking, testing for normality can be problematic (suffers from type 1 and 2 error like other statistical tests), and non-parametric only reduce power by a small amount. Therefore, I would suggest just using non-parametric tests throughout the analyses in this paper. This would simplify the interpretation and provide consistency throughout the results.

Minor Comments

1. Line 193: it is unclear what statistical programs were utilized for which analysis (JMP or GraphPad)
2. Line 198: Kruskal-Wallis is not an “analysis of variance” so calling it an ANOVA is inappropriate here
3. Line 198: I am a tad confused why the Steel-Dwass test was utilized here. This test is rarely used (in comparison to other post-hoc tests). Why use this post-hoc over the more powerful Conover-Imran, Nemenyi, or the Dunn tests?
4. Line 200: I believe I have encountered two typos. First, the Wilcoxon (not Wilcoxin) signed rank test is a one-sample or paired samples test, and I believe the authors may be referring to the Wilcoxon sum rank test (also referred to as the Mann-Whitney U-test or the Wilcoxon-Mann-Whitney sum rank test). Second, the data is not parametric or nonparametric, the *statistical tests* involve the estimation of a parameter or not.
